# Amyotrophic Lateral Sclerosis With Concurrent LHON-associated m.14484T>C Mutation: A Case Report and Literature Review

**DOI:** 10.31083/RN44110

**Published:** 2025-12-18

**Authors:** Jie-Ying Wu, Shan Ye, Tie-Lun Yin, Shuo Zhang, Dan-Feng Zheng, Jia-Yu Fu, Guang-Wei Ma, Dong-Sheng Fan

**Affiliations:** ^1^Department of Neurology, Peking University Third Hospital, 100191 Beijing, China; ^2^Beijing Municipal Key Laboratory of Biomarker and Translational Research in Neurodegenerative Diseases, 100191 Beijing, China; ^3^Department of Neurology, Xuanwu Hospital of Capital Medical University, 100053 Beijing, China; ^4^Department of Pathology, Peking University Health Science Center, 100191 Beijing, China; ^5^Peking University Sixth Hospital, 100083 Beijing, China; ^6^Key Laboratory for Neuroscience, National Health Commission/Ministry of Education, Peking University, 100871 Beijing, China

**Keywords:** amyotrophic lateral sclerosis, muscle weakness, m.14484T>C, *MT-ND6* gene, Leber’s hereditary optic neuropathy, esclerosis lateral amiotrófica, debilidad muscular, m.14484T>C, gen *MT-ND6*, neuropatía óptica hereditaria de Leber

## Abstract

**Background:**

Amyotrophic lateral sclerosis (ALS) is a rare neurodegenerative disease that mostly presents as sporadic cases. Currently, no mitochondrial-related gene mutations have been identified as the cause of ALS. Mitochondrial gene mutations cause rare hereditary diseases, and the symptoms of pure muscle weakness and muscle atrophy are rarely observed.

**Case Report:**

We report the case of a young patient clinically diagnosed with ALS concurrently associated with a pathogenic mutation in the mitochondrially encoded nicotinamide adenine dinucleotide: ubiquinone oxidoreductase core subunit 6 (*MT-ND6*) gene. However, the pathogenic relationship between the *MT-ND6* gene and ALS has not been confirmed.

**Conclusion:**

We provide a case report and a literature review aimed at increasing the understanding of the connection between the two. It is essential to consider the potential modifying role of mitochondrial pathogenic genes in ALS.

## Abstract (es)

**Antecedentes:**

La esclerosis lateral amiotrófica (ELA) es una enfermedad neurodegenerativa rara que se presenta principalmente en casos esporádicos. Actualmente, no se han identificado mutaciones genéticas relacionadas con las mitocondrias como causa de la ELA. Las mutaciones genéticas mitocondriales causan enfermedades hereditarias raras, y rara vez se observan los síntomas de debilidad muscular pura y atrofia muscular.

**Informe del Caso:**

Presentamos el caso de un paciente joven diagnosticado clínicamente con ELA asociada de forma concurrente a una mutación patógena en el gen nicotinamida adenina dinucleótido: subunidad central 6 de la ubiquinona oxidorreductasa (*MT-ND6*) codificado mitocondrialmente. Sin embargo, no se ha confirmado la relación patógena entre el gen *MT-ND6* y la ELA.

**Conclusión:**

Presentamos un caso clínico y una revisión bibliográfica con el objetivo de comprender mejor la conexión entre ambos. Es esencial tener en cuenta el posible papel modificador de los genes patógenos mitocondriales en la ELA.

## 1. Introduction

Amyotrophic lateral sclerosis (ALS) is characterized by the progressive degeneration of upper and lower motor neurons. Its typical clinical features include muscle weakness, muscle atrophy, dysarthria, and respiratory failure [1]. The peak age at onset is 58–63 years for sporadic disease and 47–52 years for familial disease [2]. While most ALS patients are classified as having sporadic ALS, up to 10% of ALS patients with a family history have familial ALS, and two-thirds carry ALS-related gene mutations [3].

Here, we report the case of a young female patient with ALS. Using mitochondrial full-genome analysis, we identified a homoplasmic variation (m.14484T>C; p. Met64Val) in the mitochondrially encoded nicotinamide adenine dinucleotide: ubiquinone oxidoreductase core subunit 6 (*MT-ND6*) gene that encodes the subunit ND6 in mitochondrial respiratory chain complex I, also known as nicotinamide adenine dinucleotide (NADH) dehydrogenase subunit 6. This mutation results in functional impairment of the mitochondrial respiratory chain, thereby affecting the process of mitochondrial energy production. ND6 is one of the NADH dehydrogenase (ND) subunits of Complex I, alongside ND1–ND5 and ND4L. Mutations in genes encoding these ND subunits are associated with classic mitochondrial disorders such as Leigh syndrome, mitochondrial encephalomyopathy, lactic acidosis, and stroke-like episodes (MELAS), myoclonic epilepsy with ragged red fibers (MERRF)-like syndromes, and Leber’s hereditary optic neuropathy plus (LHON-plus) [4, 5]. However, the clinical spectrum of Complex I deficiency extends beyond these, encompassing wide phenotypic heterogeneity, including congenital lactic acidosis, cardiomyopathy, and, rarely, ALS-like presentations.

## 2. Case Report

A 36-year-old Chinese Han woman with a 7-month history of gradually progressive distal left upper extremity weakness and atrophy as the chief complaint was admitted to the hospital. She first exhibited weakness in the distal part of her left hand 7 months prior, followed by progressive atrophy. She later noted muscle twitches, particularly in the left upper limb. At the time of admission, the other limbs remained unaffected. Motor examination revealed thenar atrophy in the left hand, first interosseous muscle, and anterior forearms. The patient denied any history of exercise intolerance or muscle pain. In her past medical history, the patient underwent craniotomy surgery for a sellar mass (5 × 5 × 5 mm^3^) at the age of 17 years due to a two-month period of painless global visual acuity decline in both eyes, and postoperative pathology indicated a simple cyst. One month after surgery, the patient’s vision had recovered to a normal level. The patient had a height of 168 centimeters and a weight of 62 kilograms, which gave her a body mass index (BMI) of 22.0 kg/m^2^. For the CARE checklist provided in **Supplementary Material-1**.

The family history was negative for neurological disorders. The patient had one sister and one brother, as well as a 13-year-old daughter and a 5-year-old son. The mother has two sisters and one brother. All of these individuals were healthy.

Strength testing revealed weakness in both upper limb movements on the Medical Research Council scale (on the left hand: finger abduction/adduction/flexion 3/5, finger extension 4/5, wrist flexion/extension 4/5, elbow flexion 4/5, elbow extension 4/5, and shoulder abduction/adduction/flexion/extension 4/5). On the right hand, her finger abduction/adduction/flexion/extension ratio was 4/5, and the strength of the other muscles was normal. Muscle strength in both lower limbs was normal. All the deep tendon reflexes were present and hyperactive, but there was no ankle clonus. Hoffmann’s and Rossolimo’s signs were present in both hands. Superficial abdominal reflexes and Babinski’s signs were absent. Cognitive, mental, cranial nerve, and sensory examinations were normal.

Routine blood test results were normal. The patient underwent lumbar puncture, and the cerebrospinal fluid (CSF) pressure was within the normal range. CSF analyses revealed normal levels of protein with no cells or oligoclonal bands. Additionally, the patient’s CSF was negative for both the ganglioside antibody spectrum and paraneoplastic syndrome antibodies.

**Fig. 1.**
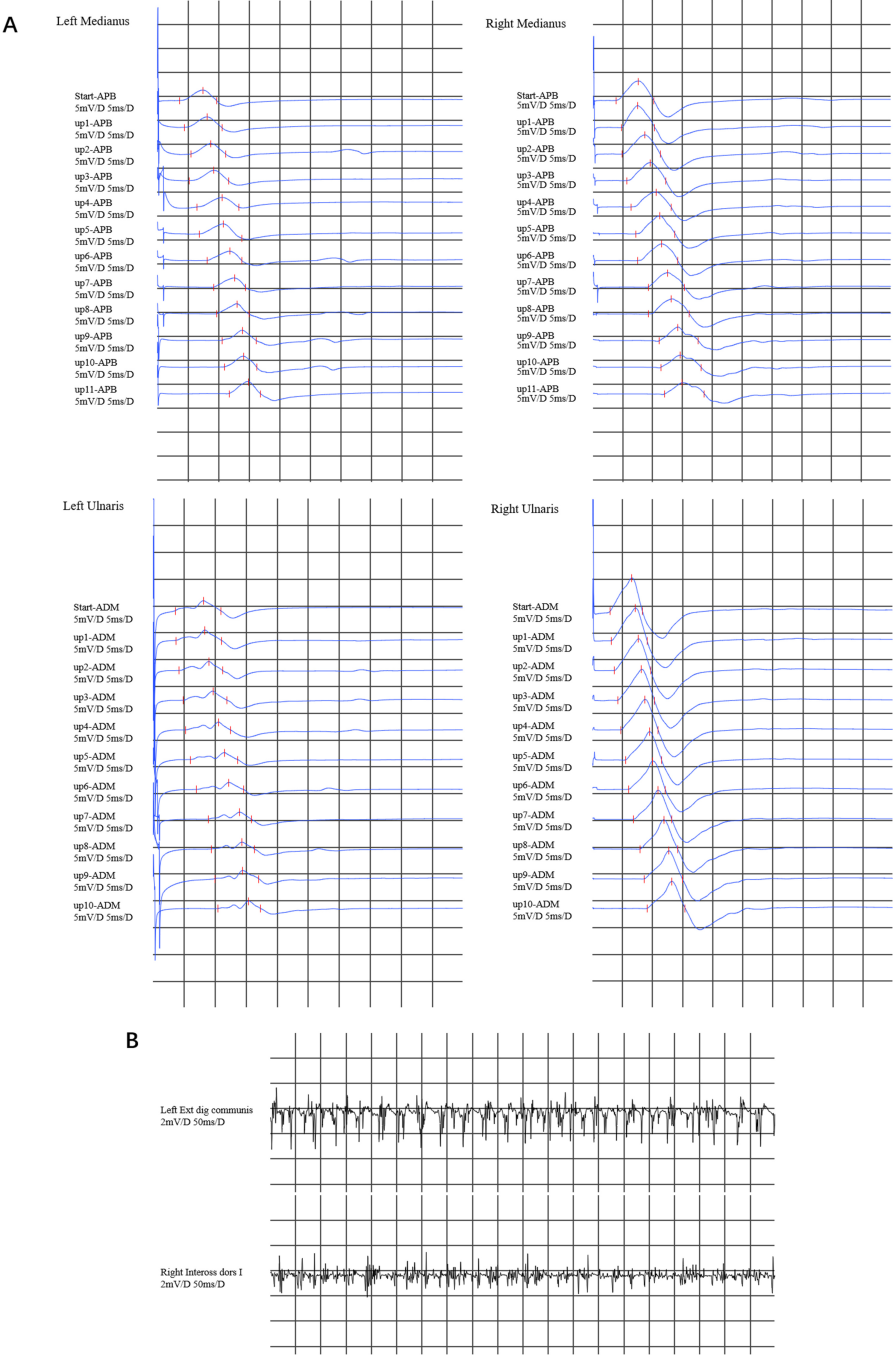
**Electromyography results**. (A) In the inching test of both the median nerve and the ulnar nerve, a decrease in compound muscle action potentials (CMAPs) was observed in the left median nerve and the ulnar nerve. The CMAP in the proximal part of the right median nerve decreased, whereas the CMAP in the right ulnar nerve was normal. There was no evidence of conduction block in any of the upper limb nerves. (B) Electromyographic (EMG) revealed that the left extensor digitorum communis muscle and right first dorsal interosseous muscle exhibited a simple mixed phase during vigorous contraction. APB, abductor pollicis brevis; ADM, abductor digiti minimi.

Nerve conduction studies revealed decreased compound muscle action potentials (CMAPs) in the left median nerve, ulnar nerve, and proximal part of the right median nerve, but we did not find conduction blockade during the inching test (Fig. 1A), with normal motor nerve conduction velocities or sensory nerve action potentials. Electromyographic (EMG) evaluation revealed high-amplitude polyphasic motor unit potentials, fibrillation and fasciculation potentials (FPs), and incomplete interference patterns, indicating neurogenic disorders in the upper (left extensor digitorum communis muscle and right first dorsal interosseous) limbs (Fig. 1B). No evidence of neurogenic damage was found in the muscles tested in the bulbar or lower limbs.

**Fig. 2.**
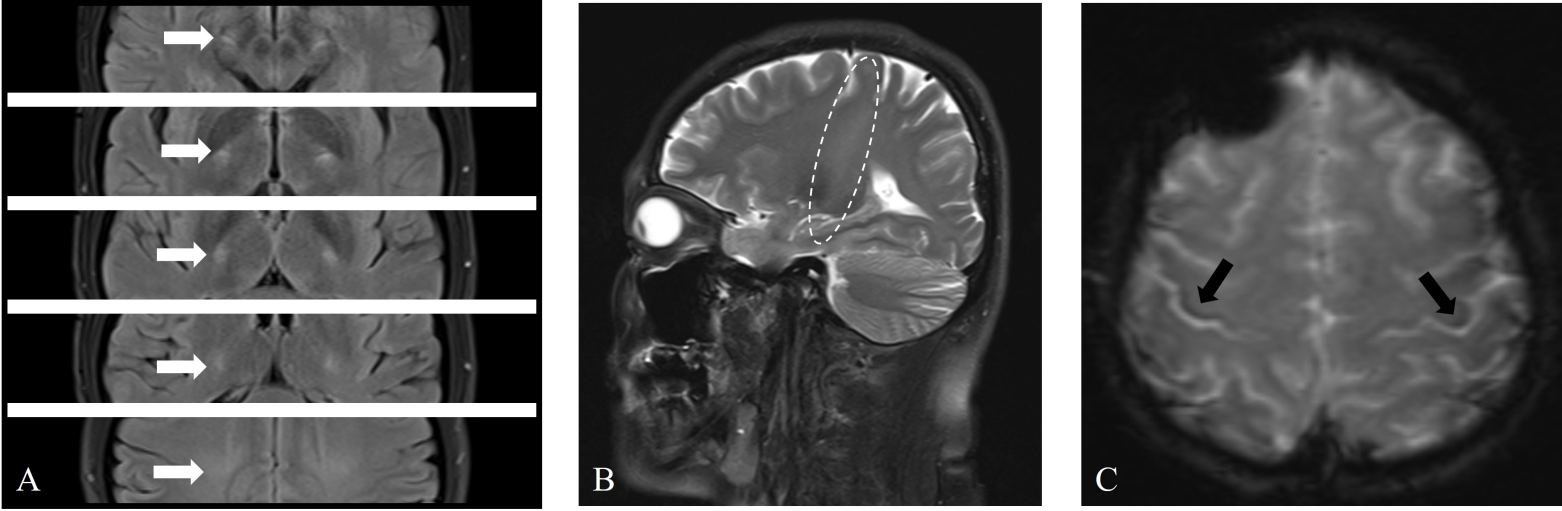
**Brain magnetic resonance imaging (MRI) revealed abnormal signals in the bilateral corticospinal tract (CST) and bilateral precentral gyrus**. (A) Axial T2 fluid attenuated inversion recovery (FLAIR) sequences showing symmetrical high signals in the CST bilaterally (white arrows). (B) Sagittal T2 sequences revealing long-segment high signals in the CST (elliptical circle). (C) Axial T2-star weighted imaging (T2*) sequences showing asymmetric curvilinear bands of low signals in the precentral gyrus (right more than left) (black arrows).

**Fig. 3.**
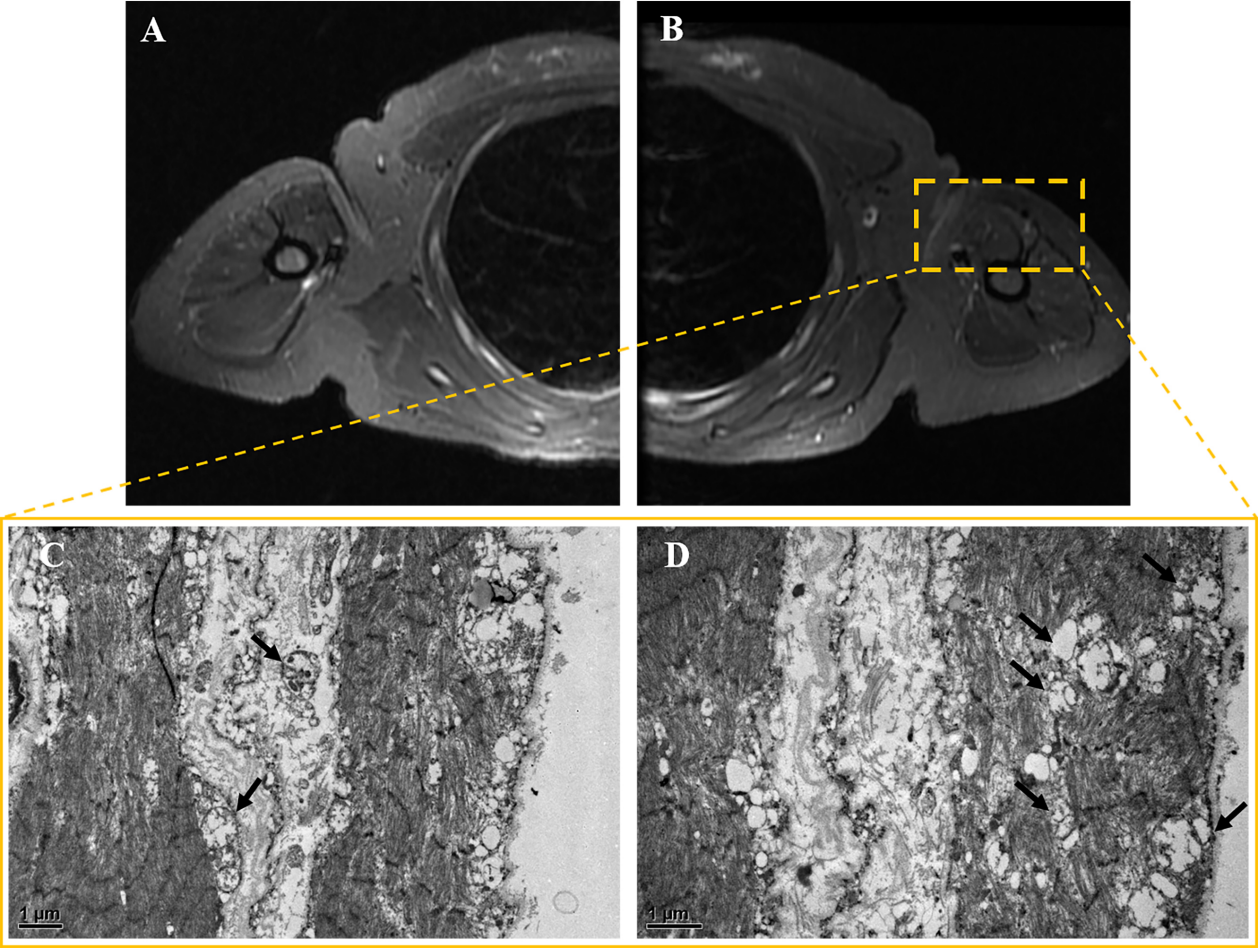
**Muscle MRI and pathology results**. (A,B) Bilateral upper limb muscle MRI. The axial short TI inversion recovery (STIR) sequence revealed no significant atrophy, hypertrophy, edema, or inflammatory changes in either upper arm. (C,D) Muscle electron microscopy images. At magnifications of (C) 12,000× and (D) 15,000×, increased mitochondrial density and swelling can be observed (black arrows). Scale bar = 1 μm.

The patient underwent a series of imaging examinations, including brain magnetic resonance imaging (MRI) (Fig. 2), cervical spine MRI, brachial plexus MRI, and bilateral upper limb muscle MRI (Fig. 3A,B). High signal intensity was observed in the corticospinal tract (CST) on T2 and T2 fluid attenuated inversion recovery (FLAIR) sequences (Fig. 2A,B), and hypointensity was observed in the bilateral posterior part of the precentral gyrus on T2-star weighted imaging (T2*) sequences, known as the motor band sign (MBS) (Fig. 2C). MRI of other areas was normal. Additionally, ultrasonic cardiography, 24-hour Holter monitoring, and abdominal ultrasound revealed no significant abnormalities. The pulmonary function test indicated that the forced vital capacity was 113.6% of the predicted value.

Muscle biopsy was performed on the patient’s left biceps brachii muscle. These pathological findings suggest that approximately 30%–50% of muscle fibers exhibit bundle atrophy accompanied by compensatory hypertrophy. Muscle fiber grouping was observed via NADH staining. Modified Gomori Trichrome (MGT) staining revealed no typical ragged-red fibers (RRFs). Cytochrome oxidase (COX) and succinate dehydrogenase (SDH) staining revealed the deposition of small amounts of subsarcolemmal material in some muscle fibers (see** Supplementary Material-2**). No definite infiltration of inflammatory cells was observed. Electron microscopy revealed mitochondrial proliferation in some muscle fibers and abundant swollen mitochondria beneath the sarcolemma in a few fibers (Fig. 3C,D). Overall, the pathological diagnosis was consistent with moderate neurogenic muscle atrophy with mitochondrial abnormalities.

Peripheral blood and muscle samples were collected from patients at Peking University Third Hospital. Whole-exome sequencing, mitochondrial gene testing, and multiplex ligation-dependent probe amplification (MLPA) analysis of the peripheral myelin protein 22 (*PMP22*) gene were performed by Beijing Kangso Medical Inspection Co., Ltd. Additionally, Sanger sequencing of the identified abnormal genes was performed.

Mutational screening was negative for all known pathogenic genes except for mitochondrial DNA (mtDNA) *MT-ND6* (m.14484T>C), which revealed 99.41% homoplasmic variation. This allele changes the weakly conserved methionine at amino acid 64 to valine (p. Met64Val). This mutation is one of the three common mutation sites in Leber’s hereditary optic neuropathy (LHON) disease (the other two common pathogenic mutations are m.11778G>A in the *MT-ND4* gene and m.3460G>A in the *MT-ND1* gene) [6]. A mutation in the *MT-ND6* gene has also been identified in a small number of people with Leigh syndrome [7]. A comprehensive ophthalmologic examination of the patient revealed normal visual acuity and a normal fundus. Additionally, optical coherence tomography (OCT) revealed no abnormalities.

**Fig. 4.**
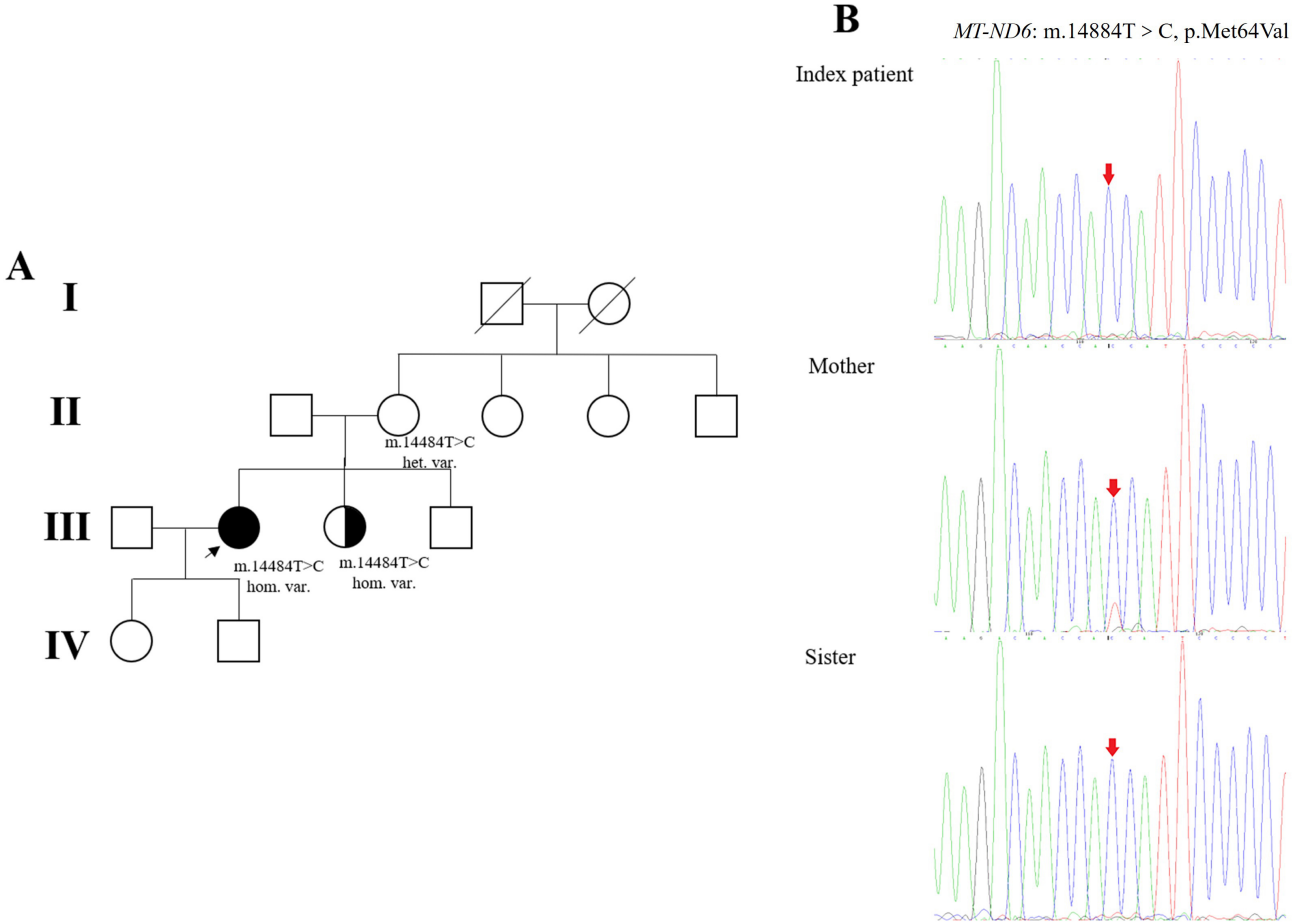
**Pedigree of the patient’s family and genetic analysis**. (A) The proband is indicated by an arrow. Males and females are represented as squares and circles, respectively. The patients’ parents, siblings, and children were all healthy. The patient is indicated by black filled symbols, individuals with asymptomatic homoplasmic m.14484T>C mutations are indicated by a black semicircle symbol, and unfilled symbols indicate unaffected individuals. (B) The proband has a homoplasmic mutation for the pathogenic variant p.Met64Val in the mitochondrially encoded nicotinamide adenine dinucleotide: ubiquinone oxidoreductase core subunit 6 (*MT-ND6*) gene. The proband’s mother had a heteroplasmic mutation, whereas her sister had a homoplasmic mutation. The red arrow indicates the mitochondrial mutation site identified in the proband (index patient), her mother, and her sister. Both the proband and her sister carried this variant homoplasmically, whereas their mother exhibited a heteroplasmic state.

The *MT-ND6* gene was screened from her asymptomatic mother and sister (Fig. 4A). The mother presented heteroplasmic variation at the same site, whereas the younger sister presented homoplasmic variation (Fig. 4B). Currently, the patient’s sister has no symptoms of visual impairment or muscle weakness.

The results of the mitochondrial gene analysis of muscle samples also revealed homoplasmic variation in the *MT-ND6* gene at position m.14484T>C, with a heterozygosity rate of 99.81%.

In addition to the LHON-associated mutation (homoplasmic m.14484T>C/*MT-ND6* in this patient), complete mtDNA sequencing revealed a second homoplasmic m.12338 T>C/*MT-ND5* variant, which was detected in the patient’s blood and muscle tissue. This locus has also been reported in LHON, but its pathogenicity has not yet been clearly established [8, 9].

The next-generation sequencing (NGS) panel revealed variants of interest in this patient. She carried the c.5225T>A, p.Leu1752Gln heterozygous variant in the kinesin family member 1B (*KIF1B*) gene (OMIM#118210, phenotype: Charcot-Marie-Tooth disease (CMT), autosomal dominant). The variant is classified as likely pathogenic on the basis of relevant clinical and laboratory data. Her father is also a carrier of a heterozygous variant in this gene. Owing to the lack of cosegregation of this gene, it can be excluded as a causative gene. Additionally, this patient carried the c.1205T>A and p.Ile402Asn heterozygous variants in the F-box protein 38 (*FBXO38*) gene (OMIM#615575, phenotype: distal hereditary motor neuropathy, autosomal dominant) and the c.358T>G and p.Leu120Val heterozygous variants in the tropomyosin-receptor kinase fused (*TFG*) gene (OMIN#615658, phenotype: hereditary paraplegia, autosomal recessive); her mother and father had heterozygous variants in these two genes. These two variants are novel and are classified as uncertain in significance with minor pathogenic evidence according to the American College of Medical Genetics (ACMG) classification.

We excluded other mitochondrial syndromes (such as MELAS, Leigh, MERRF-like, and LHON-plus) on the basis of clinical presentation (absence of exercise intolerance, myoclonus, etc.), muscle biopsy (absence of ragged-red fibers), and brain imaging (absence of cortical or white matter lesions and normal structure of the visual pathways). At the time of clinical diagnosis, the young woman presented with pure motor involvement and met the revised El Escorial and Gold Coast criteria [10, 11]; this condition was confirmed as ALS (clinical-instrumental results are shown in Table 1). Additionally, on the basis of the patient’s genetic results along with her history of painlessness and progressive bilateral vision loss, she was confirmed to have LHON disease.

**Table 1.** **Results of the neurological workup**.

Items	Available evidence
Neurological examination	LMN signs at cervical region, UMN signs at upper and lower limbs
EMG	LMN sign in one region (upper limbs)
Blood and CSF exams	Unremarkable
Clinical diagnosis	Laboratory-supported probable (El Escorial criteria)

Abbreviations: LMN, lower motor neuron; UMN, upper motor neuron; EMG, electromyography; CSF, cerebral spinal fluid.

We mainly employ cocktail therapy for treating mitochondrial diseases, which primarily utilizes medications for energy and vitamin supplementation, and riluzole is used to treat ALS. Additionally, adequate nutrition and weight maintenance are essential. Regular evaluations to detect manifestations that can occur with time include neurologic deficits, psychiatric abnormalities, impaired respiratory function, and loss of vision.

**Fig. 5.**
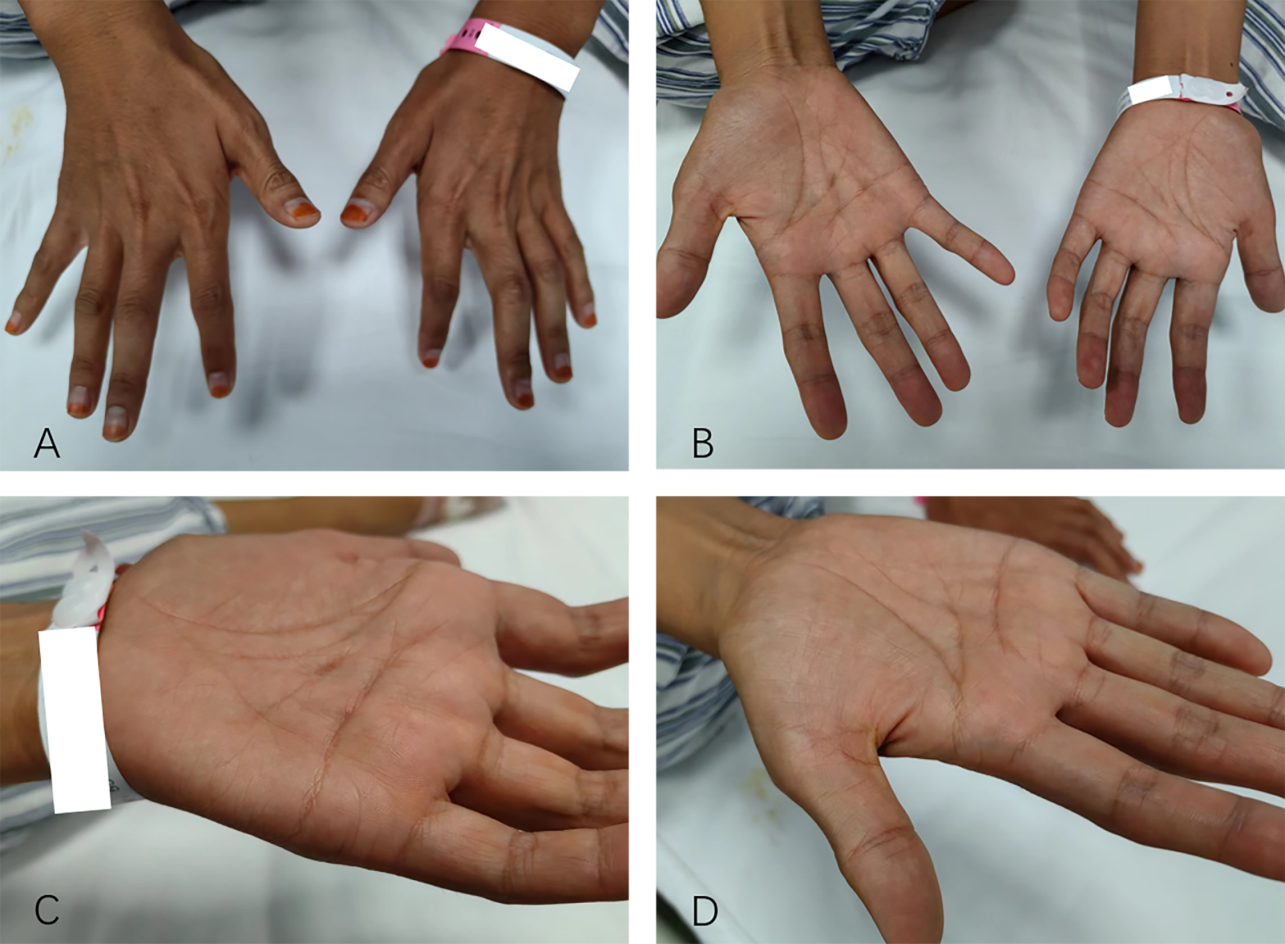
**The patient had atrophy in the thenar muscles of both hands**. (A) Back of both hands. (B) Palms of both hands. (C) Palm of the left hand. (D) Palm of the right hand.

Three months after the follow-up, the patient reported a reduction in muscle fasciculations compared with before, but weakness in the right hand had also emerged. Over time, the weakness and atrophy in both hands gradually worsened (Fig. 5). The patient’s timeline of symptom onset and progression is illustrated in the** Supplementary Material-2**.

## 3. Discussion

Primary mitochondrial diseases are a group of inherited metabolic disorders caused by mutations in mtDNA or nuclear DNA [12]. Diseases associated with mtDNA mutations exhibit significant clinical heterogeneity, impact isolated or multiple organ systems, and can manifest at any age [13]. Furthermore, these diseases do not follow Mendelian inheritance.

Although ALS is generally considered a single disease entity, there are various classifications based on genetic and phenotypic patterns, and it is likely that it is more appropriate to consider this a syndrome of motor neuron degeneration with multiple causes.

In this report, the homoplasmic m.14484T>C (p.Met64Val) variant of the *MT-ND6* gene was shown to coexist with early-onset ALS in an individual. The m.14484T>C/*MT-ND6* gene mutation is a common pathogenic variant in LHON, and there have been no previous reports of its association with ALS at the same locus. Currently, reports on the relationship between mitochondrial gene variations and ALS are limited. The association between ALS and a mutation at another common pathogenic locus in LHON, the m.11778A>G/*MT-ND4* gene, has been described [14]. This report revealed that two patients with the mutation who were aged 73 and 74 years developed symptoms of ALS. Although the co-occurrence of mitochondrial gene mutations and ALS has been considered, whether mitochondrial gene mutations play a modifying role in ALS remains to be determined. Compared with other patients, our patients had an earlier onset age, suggesting the potential early modifying effect of mitochondrial gene mutations.

We tested the patient’s sister and confirmed that she carried the homoplasmic mutation in her mtDNA at the same position. Although she is asymptomatic, the penetrance of optic neuropathy in the Chinese LHON family ranges from 5.6% to 100% [15], and we suggest that this mutation can exhibit incomplete penetrance and variable expressivity. The impact of the mutation may be regulated or modified by other factors, including the influence of other genes (such as nuclear genes) and environmental factors, even mitochondrial haplogroups. These factors determine the level of phenotypic penetrance and the affected tissues, which in turn may determine the inheritance pattern of the disease as well as its onset and progression [16]. In this scenario, we assume that the patient and her sister share similar environmental factors. However, on the basis of nuclear genetic inconsistencies, factors potentially affecting mtDNA stability, energy metabolism, or repair mechanisms, thereby influencing disease manifestation, even though we currently have not identified any pathogenic mutations in the nuclear genes. Some mitochondrial diseases exhibit incomplete penetrance due to the influence of haplogroups. However, a single study of 700 patients and 462 controls in the European population did not reveal any association between mitochondrial haplogroups and ALS, suggesting that mitochondrial DNA haplogroup variations may not be the primary genetic risk factor for ALS [17]. Additionally, previous study has suggested that there is no significant correlation between the level of heteroplasmy associated with primary mtDNA LHON mutations and the severity of the clinical phenotype or the risk of visual loss [15]. These mutations may not have a deleterious synergistic effect.

According to LHON cohort analysis in China, all patients carried the m.14484T>C mutation, but there were different mtDNA polymorphisms [15]. The presence of another m.12338T>C/*MT-ND5* homoplasmic mutation in our patient may have enhanced the penetrance of vision loss. Additionally, the patient carried the c.5225T>A, p.Leu1752Gln heterozygous variant in the *KIF1B* gene, which is considered a potentially pathogenic gene associated with CMT. This scenario of multiple variants in the nuclear and mitochondrial genomes possibly contributing to multilayered mitochondrial dysfunction highlights the complexities of the genetic background in sporadic ALS. Mitochondrial dysfunction may occur due to mutations in mtDNA and their association with mutations in various genes contributing to neurodegenerative disorders.

The MitPhen database (http://www.mitophen.org/) [18] of pathogenic mtDNA genes and human phenotypic ontologies (HPOs) has been established. Among the 111 mtDNA mutations, 89 met the pathogenicity criteria (4 insertions and deletions, 85 single nucleotide variants (SNVs)), 40 of which were located in the mtDNA coding region. The total number of pathogenic mutations covered 26,348 HPOs. In the MitoPhen database 1.7, a total of 530 MitoPhen patients carrying the m.14484T>C mutation were identified. By searching for the term “muscle weakness” in the HPO terms, we found 11 individuals from 10 pedigrees (see Table 2). Among them, “lower limb muscle weakness” was observed in 5 patients, “proximal muscle weakness in the lower limbs” in 2 patients, and “distal muscle” in 4 patients. Additionally, one patient experienced both “upper limb muscle weakness” and “lower limb muscle weakness”. Some patients presented other evidence supporting ALS, such as “lower limb hyperreflexia”, “hyperreflexia in upper limbs”, “areflexia of lower limbs”, “electromyography (EMG): neuropathic changes”, and “Babinski sign”. On the basis of these data, the m.14484T>C mutation can lead to impaired motor function. However, some patients presented evidence not supporting ALS, including “presthesia”, “abnormality of peripheral somatosensory-evoked potentials”, “distal sensory impairment”, “sensory axonal neuropathy”, “impaired vibration sensation in the lower limbs”, “impaired distal tactile sensation”, “back pain”, and “episodic pain”. We did find that sensory symptoms or signs can indeed occur in many classic ALS patients [19].

**Table 2.** **According to the Human Phenotype Ontology (HPO) database, individuals carrying the m.14484T>C mutation exhibit muscle weakness**.

Family id	Sex	Onset age	Variant	Tissue	Variant presence	Heteroplasmy	PMID	Symptoms of motor system	Supported signs	Signs or symptoms of sensory system	Diagnosis
10414484Fa	F	32	m.14484T>C	Blood	Present	Null	18344382	Lower limb muscle weakness; Upper limb muscle weakness	Lower limb hyperreflexia; Hyperreflexia in upper limbs	Null	Harding’s syndrome
1914484Ma	M	15	m.14484T>C	Blood	Homoplasmic	100	21685233	Lower limb muscle weakness	Null	Paresthesia; Abnormality of peripheral somatosensory evoked potentials	Harding’s syndrome
2114484Ma	M	6	m.14484T>C	Blood	Homoplasmic	100	29249004	Proximal muscle weakness in lower limbs	Areflexia of lower limbs	Distal sensory impairment	Leber hereditary optic neuropathy and longitudinally extensive transverse myelitis
2214484Fa	F	64	m.14484T>C	NG	Present	Null	21734595	Lower limb muscle weakness	EMG: neuropathic changes	Sensory axonal neuropathy	Leber hereditary optic neuropathy
2414484Ma	M	33	m.14484T>C	Blood	Homoplasmic	100	27486939	Proximal muscle weakness in lower limbs	Lower limb hyperreflexia	Impaired vibration sensation in the lower limbs; Impaired distal tactile sensation; Abnormality of peripheral somatosensory evoked potentials	Leber’s ‘Plus’
3214484Ma	F	15	m.14484T>C	Blood	Homoplasmic	100	8470982	Distal muscle weakness	Null	Paresthesia	Leber hereditary optic neuropathy
3214484Ma	M	17	m.14484T>C	Blood	Homoplasmic	100	8470982	Distal muscle weakness	Null	Paresthesia	Leber hereditary optic neuropathy
3314484Ma	M	33	m.14484T>C	Blood	Homoplasmic	100	8470982	Distal muscle weakness	Null	Paresthesia	Leber hereditary optic neuropathy
4714484Fa	F	21	m.14484T>C	Blood	Homoplasmic	100	8470982	Distal muscle weakness	Null	Paresthesia	Leber hereditary optic neuropathy
9414484Ma	M	36	m.14484T>C	Blood	Present	Null	11450909	Lower limb muscle weakness	Null	Back pain	Leber hereditary optic neuropathy
9814484Fa	F	18	m.14484T>C	Blood	Homoplasmic	100	15483043	Lower limb muscle weakness	Babinski sign	Paraparesis; Episodic pain	White matter disease in Leber’s hereditary optic neuropathy

Abbreviations: F, female; M, male; NG, not given; PMID, PubMed unique identifier.

Recently, a large study on ALS pointed to the burden of multiple risk factors identified in the nuclear genome, but the impact of mtDNA variation was not considered [20]. We did not find any mtDNA-related information associated with ALS in the ALS Online Database (https://alsod.ac.uk/). In our case, the patient presented with young-onset ALS in the context of a confirmed diagnosis of mtDNA-related disorder. Although we cannot definitively confirm that this mtDNA site is the causative gene for ALS, it is worth considering that the mutation at this gene site may contribute to the early onset of ALS and confer genetic risk.

Frameshift mutations in genes encoding mitochondrial respiratory chain complex I have previously been reported to occur in individuals with ALS, but such mutations are rare [21]. Mutations in the nuclear genecoiled-coil-helix-coiled-coil-helix domain containing 10 (*CHCHD10*) are pathogenic mutations in ALS, and *CHCHD10* is a mitochondrial protein located in the intermembrane space. This gene mainly causes mtDNA instability disorders through the accumulation of multiple mtDNA deletions, but these mutations are mainly responsible for the clinical spectrum of frontotemporal dementia (FTD)-ALS [22]. In addition, other rarer mutations that affect mtDNA instability, such as DNA polymerase subunit gamma-1 (*POLG*), thymidine kinase 2 (*TK2*) or deoxyguanosine kinase (*DGUOK*), can cause ALS-like symptoms [23, 24, 25]. This evidence suggests that mitochondrial diseases may be the origin of some phenotypes of ALS, opening a new field in which to explore the pathogenesis of the clinical spectrum of ALS.

Research on ALS patients has revealed the following factors: the accumulation of mitochondria in proximal axons, mitochondrial injury caused by excessive reactive oxygen species (ROS), *COX I* mtDNA mutation, and RRF. These factors act mainly through increased ROS and altered mitochondrial structure [16]. mtDNA deletions are more common in individuals with sporadic ALS than in healthy controls [26]. In sporadic ALS patients, the presence of COX-negative muscle fibers in skeletal muscles is common, but no correlation has been found between the severity of oxidative defects and patient age or disease duration [27, 28].

ALS as a type of neuromuscular disorders (NMDs). There is evidence that any defects at the mitochondrial level could jeopardize the function of cells and tissues, forming the basis of NMDs [16]. Interestingly, mitochondria can also play a secondary role in the development of the remaining NMDs when the mutation or deficiency is not directly related or located in the mitochondria, since affected cells need additional adenosine triphosphate (ATP) to support homeostatic mechanism imbalance (antistress or antioxidant responses) while minimizing the production of ROS. If mitochondria are unable to counterbalance cell dysfunction, a secondary mitochondrial disease, such as ALS caused by a mutation in trans-activation response DNA-binding protein 43 (TDP-43) [29], can also occur in spinal muscular atrophy (usually caused by a mutation in the coding sequence of survival of motor neuron 1) [30]. Therefore, disregarding genetic origins, mitochondrial function is key in the onset or progression of most NMDs.

From a genetic variation perspective, impaired Complex I function increases electron leakage during the electron transfer process, leading to elevated production of ROS, which constitute one of the pathogenic factors in ALS. Additionally, mitochondrial dysfunction disrupts mitochondrial dynamics (including fission, fusion, and transport), thereby impairing axonal transport [31]. For example, in drosophila models [32], loss of mitochondrial Complex I causes mitochondrial transport defects characterized by drastically reduced velocity and flux of mitochondrial movement within axons.

However, research on the relationship between the m.14484T>C mutation in the *MT-ND6* gene and ALS remains limited. On the basis of this case report, additional studies are needed to elucidate how mtDNA mutations may be linked to both monogenic and sporadic ALS, and larger sample sizes are needed in future research to verify these findings. Moreover, we must acknowledge the possibility that the simultaneous occurrence of ALS and the *MT-ND6* mutation in this case may be coincidental, with no causal relationship existing between them.

## 4. Conclusion

In conclusion, we report the case of an ALS patient with concurrent LHON disease. Her m.14484T>C homoplasmic mutation is the first such mutation to be reported in ALS patients.

## Availability of Data and Materials

Study data are available from the corresponding author upon request.

## Supplementary Material

Supplementary material associated with this article can be found, in the online version, at https://doi.org/10.31083/RN44110.
