# Supplementary material for: Amyotrophic Lateral Sclerosis With Concurrent LHON-associated m.14484T>C Mutation: A Case Report and Literature Review
Source: Rev Neurol. 2025 Dec 18;80(11):44110. doi: 10.31083/RN44110 (PMC12781225; doi:10.31083/RN44110)
Supplement: Supplementary file 1 [file 1576-6578-80-11-44110-s1.zip › Supplementary Material-2.docx]

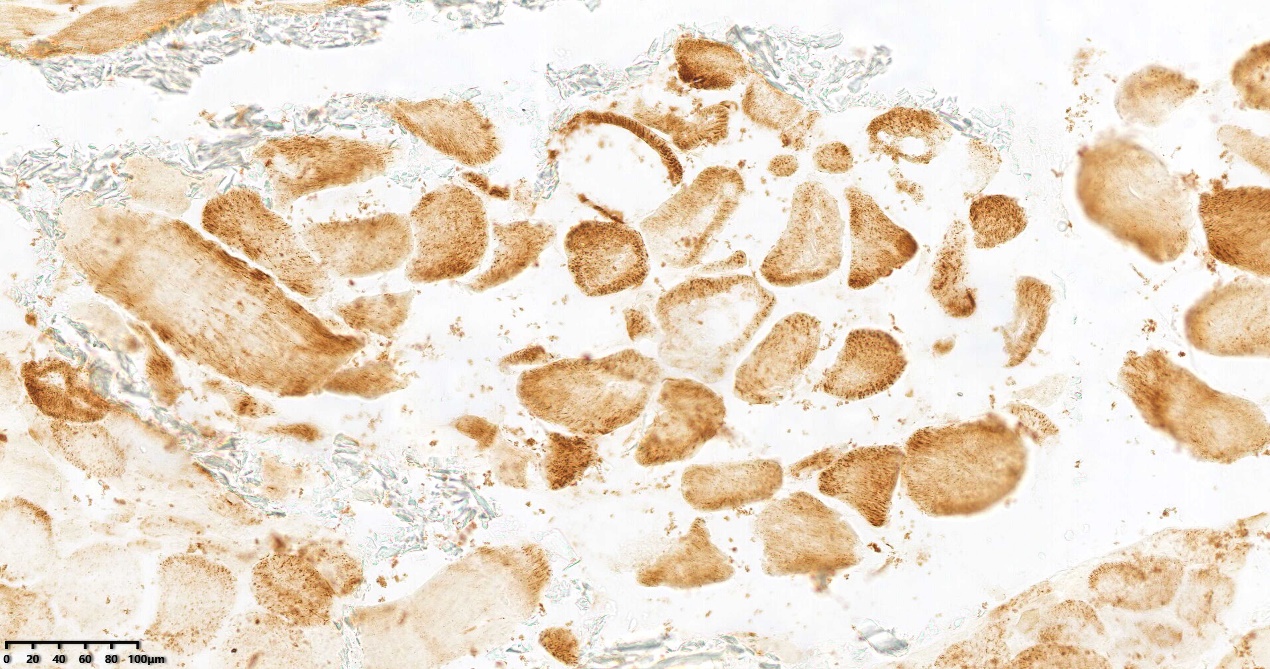


Supplementary Figure 1 Muscle biopsy COX stain showing mild subsarcolemmal deposits


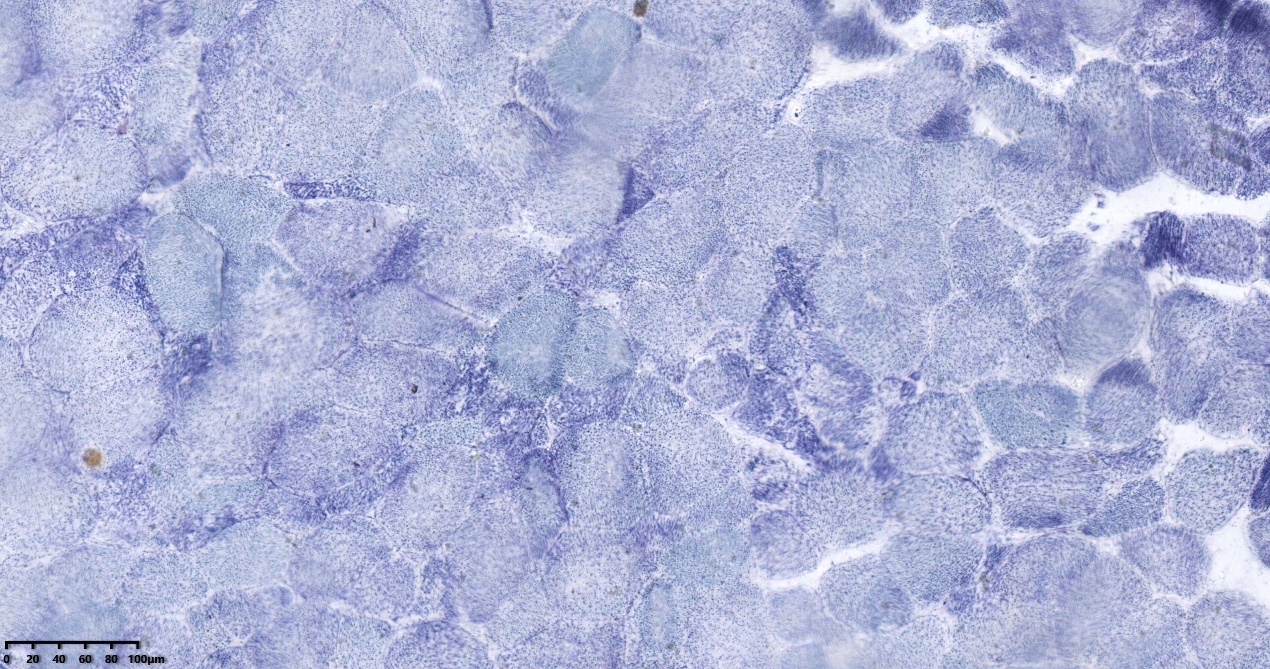


Supplementary Figure 2 Muscle biopsy SDH stain also showing mild subsarcolemmal deposits


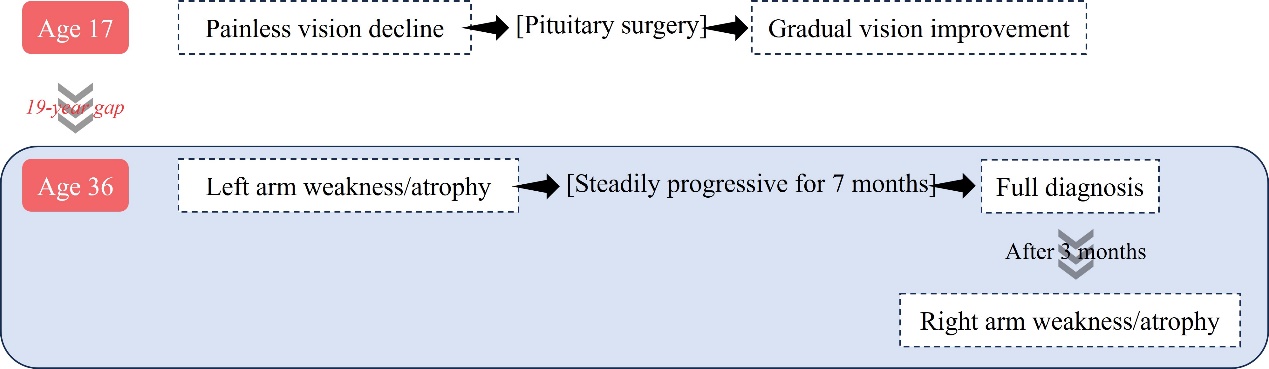


Supplementary Figure 3 Timeline summarizing the patient's disease onset, symptom progression, investigations, and diagnosis
